# Building research capacity to adapt and develop Patient-Reported outcome measures in low- and middle-income countries: results from a psychometrics workshop in Tanzania

**DOI:** 10.1186/s12913-025-13064-2

**Published:** 2025-07-01

**Authors:** Berivan Ece, Mrema Kilonzo, Sylvia Kaaya, Edith Tarimo, Candida Moshiro, Erasto Mbugi, Linda B. Mlunde, Lisa R. Hirschhorn, Claudia Hawkins, Emily H. Ho

**Affiliations:** 1 Department of Medical Social Sciences, Feinberg School of Medicine, Northwestern University, 625 N. Michigan Ave., 27th floor, Room 2745, Chicago, IL 60611 USA; 2 Department of Psychiatry and Mental Health, School of Clinical Medicine, Muhimbili University of Health and Allied Sciences, Dar es Salaam, Tanzania; 3 Department of Nursing Management, School of Nursing, Muhimbili University of Health and Allied Sciences, Dar es Salaam, Tanzania; 4 Department of Epidemiology and Biostatistics, School of Public Health and Social Sciences, Muhimbili University of Health and Allied Sciences, Dar es Salaam, Tanzania; 5 Department of Biochemistry and Molecular Biology, School of Medicine, Muhimbili University of Health and Allied Sciences, Dar es Salaam, Tanzania; 6 Department of Community Health, School of Public Health and Social Sciences, Muhimbili University of Health and Allied Sciences, Dar es Salaam, Tanzania; 7 Ryan Family Center for Global Primary Care, Feinberg School of Medicine, Robert J Havey Institute for Global Health, Northwestern University, Chicago, IL USA; 8 Robert J Havey Institute for Global Health - Center for Global Communicable and Emerging Infectious Diseases, Feinberg School of Medicine, Northwestern University, Chicago, IL USA; 9 Department of Medicine, Feinberg School of Medicine, Northwestern University, Chicago, IL USA

**Keywords:** Patient-Centered outcomes research (PCOR), Tanzania, Patient-Reported outcome measures (PROMs), HIV, Research capacity-building

## Abstract

**Background:**

Effective antiretroviral treatment has transformed HIV into a manageable chronic condition. In Tanzania, about 1.7 million people are living with HIV (PLWH), with 79% achieving viral suppression. Patient-Reported Outcome Measures (PROMs) evaluate key outcomes that matter most for PLWH and are an essential tool to evaluate and improve health system performance. Research is needed to adapt and test PROMs to increase their use in HIV care in many low- and middle-income countries including Tanzania. Since 2019, Northwestern University and Muhimbili University of Health and Allied Sciences (MUHAS) have led an NIH-funded Fogarty D43 grant to build Patient-Centered Outcomes Research (PCOR) capacity in Tanzania. We report on the results of a workshop to strengthen capacity in PROMs development, adaptation and psychometric validation for improving health outcomes for PLWH in Tanzania.

**Methods:**

The three-day in-person workshop featured expert-led instruction and practical exercises on PROMs selection, development, adaptation, validity testing, factor analysis, and manuscript writing. We applied Kirkpatrick Framework (Level 1-reaction, Level 2-knowledge, and Level 3-use) for workshop evaluation. Participants completed pre-workshop surveys on goals, experience, confidence, and knowledge, followed by a post-workshop survey to assess satisfaction (Level 1), knowledge changes (Level 2), and plans for use (Level 3), along with feedback.

**Results:**

Twenty-seven participants (44.4% male) attended the workshop, with 92% stating it met workshop goals and 87.5% reporting personal goal fulfillment (Level 1). There was a significant increase in knowledge and confidence ratings across all topics (*p* <.001) (Level 2). Knowledge ratings rose from a mean of 0.44 (SD = 0.68) to 2.55 (SD = 0.56) while confidence increased from 0.47 (SD = 0.72) to 2.55 (SD = 0.56). Correct answer rates on knowledge assessments also improved and all attendees reported plans to use the skills in future research. Qualitative feedback indicated high enthusiasm for the course and increased confidence in applying the learned skills to current or future research (Level 3).

**Conclusions:**

The workshop effectively strengthened capacity of attendees to adapt and validate PROMs for use in research and care in Tanzania. Plans are in place to incorporate this training into formal MUHAS courses. Similar workshops are needed to further enhance PROMs research capacity in the region for future application both in research and clinical care of PLWH.

**Supplementary Information:**

The online version contains supplementary material available at 10.1186/s12913-025-13064-2.

## Background

Advances in care and treatment have dramatically changed HIV infection from a rapidly fatal to a manageable chronic condition. In Tanzania, about 1.7 million people are living with HIV (PLWH), with 79% achieving viral suppression [1, 2]. This transformation calls for a shift towards people-centered care, highlighting the importance of measuring and improving outcomes beyond viral suppression and ensuring that care is designed to engage and meet the evolving needs of PLWH [3]. Making care in a more people-centered manner means ensuring that services are respectful, responsive, and acceptable to those receiving them [4–6]. Incorporating these principles in HIV care and measuring them through Patient-Reported Outcomes (PROs) will help meet the diverse healthcare needs of PLWH and support their overall well-being.

The increasing focus on people-centered care for PLWH emphasizes the importance of Patient-Centered Outcomes Research (PCOR) in improving health outcomes. By prioritizing patient perspectives, PCOR methodologies facilitate the evaluation and enhancement of healthcare services. Particularly, Patient-Reported Outcome Measures (PROMs) are vital for identifying the most important outcomes for PLWH. Interest in applying PROMs in HIV research and care is growing [7, 8]. However, despite evidence of the importance of using PROs as research outcomes and integrating them into healthcare systems to improve patient satisfaction and treatment effectiveness, there remains a notable gap in their implementation in low- and middle-income countries (LMICs) [9, 10].

Although PROs include incorporating patients’ perspectives into healthcare decisions, implementing these measures across various cultures presents significant challenges [11–13]. Barriers to the uptake of PROs are prevalent in countries like Tanzania, primarily due to the limited availability of tools specifically developed or adapted for the region [14]. There are significant gaps in the skills required for the development, adaptation, and validation of these measures. Simply translating measures linguistically is not sufficient, as cultural nuances may vary widely between regions [15–17]. For instance, the Swahili used in Kenya and Tanzania may differ, necessitating careful cultural adaptation beyond mere translation [18]. To overcome these challenges, it is crucial to provide training on the cultural adaptation of measures and emphasize the importance of psychometric analyses of adapted measures [11, 19]. Investing in capacity-building in PCOR is essential for strengthening people-centered HIV care, improving quality of life and health outcomes in PLWH and achieving global UNAIDS 95 95 95 targets. By investing in PCOR training and capacity building, LMICs can significantly enhance their ability to deliver comprehensive and effective HIV care, ultimately leading to better health outcomes and improved quality of life for PLWH [20–22].

In Tanzania, there are limited opportunities for systematic training in PCOR, which is crucial for building capacity in this under-resourced setting [23]. The lack of formal training programs hinders the ability of researchers and healthcare professionals to effectively utilize PCOR methodologies to develop and assess culturally sensitive patient-centered measures, interventions, and outcomes. To address this gap, we designed and conducted a workshop with the purpose of strengthening capacity in PROMs psychometric validation and cultural adaptation in the scientific community in Muhimbili University of Health and Allied Sciences (MUHAS). The workshop was organized and facilitated by skilled researchers from Northwestern University and MUHAS, who have established expertise in psychometrics, patient-reported outcomes (PROs), and the creation and execution of patient-reported outcome measures (PROMs). Workshop training objectives and learning outcomes are presented in Table 1. We report on the effectiveness of this training based on the pre- and post-workshop evaluations by the attendees on several aspects such as their knowledge and confidence in the topics covered.

**Table 1:** Workshop training objectives and learning outcomes

**Training objectives**
1. Understanding Assessment Instruments: • To understand how assessment instruments are developed and used. • To identify the questions that need to be answered before an instrument is deemed ready for use in a new context.
2. Instrument Selection and Evaluation: • To demonstrate the process of selecting standardized assessment instruments. • To evaluate the strengths and weaknesses of selected instruments.
3. Linguistic and Cultural Adaptation: • To teach attendees how to perform linguistic and cultural adaptation for tools validated in other settings, languages, or populations.
4. Psychometric and Assessment Principles: • To demonstrate the analysis of basic psychometric and assessment principles. • To apply these principles to adaptations of research assessment instruments measuring psychosocial attributes or other health-relevant outcomes. • To interpret results of psychometric analyses or testing.
5. Understanding Published Research: • To develop trainees’ understanding of published research using assessment instruments.
6. Ethical and Multicultural Awareness: • To increase trainees’ understanding of the ethical issues surrounding patient-reported outcomes and other validated survey questionnaires. • To increase awareness of multicultural issues surrounding psychological-health measures.
**Learning outcomes**
1. Psychometric Evaluation: • Critically evaluate psychological and health measures based on their psychometric merits.
2. Adaptation Planning: • Plan for linguistic and cultural adaptation of validated tools for use in new settings and populations.
3. Research Skills: • Evaluate current research on psychological and health assessment instruments related to trainee’s own planned research/clinical assessment interests.
4. Psychometric Knowledge: • Have a working knowledge of psychometric theory and the psychometric qualities of tests. • Interpret results and collaborate to plan future analysis.
5. Ethical and Multicultural Appreciation: • Appreciate and understand ethical issues surrounding psychological and health measures.

## Methods

### Workshop and setting

A three-day in-person workshop was designed and led by experienced researchers from Northwestern University and MUHAS with recognized expertise in psychometrics, PROs, and the development and implementation of PROMs. The primary goal was to equip participants with skills and knowledge that could be applied to their ongoing and future work in PROMs and other patient-centered outcomes research, particularly in the fields of HIV. The attendees had various roles such as researchers, academicians, and clinical care providers. The workshop in PROs focused on the practical skills needed to develop and validate psychometric tools for research. The workshop was structured to cover the following topics through adult learning pedagogy including lectures, interactive discussions, and hands-on activities: PROMs selection, development, adaptation, cultural adaptation, testing for validity, factor analysis, and manuscript writing (see Supplementary Material 1 for course agenda). The interactive component of the workshop included both individual and group activities. Participants first completed structured exercises for individual practice, followed by group discussions. They were also given time to independently create questions relevant to their field, professional role, and experience level, which they then discussed in small groups. Each group presented their work to the full class at the end of the session. For example, participants worked in small groups to translate well-established measures into their local language. After translating items individually, they compared translations and discussed the implications of any differences identified.

### Participants

To be eligible to attend the workshop, applicants were required to have at least a master’s degree and experience in or plans for using PROMs in research or clinical work. Participants were recruited through advertisements on the MUHAS and National Institute for Medical Research (NIMR) web pages, as well as through word-of-mouth referrals within government institutions seeking for help in psychometrics from staff at MUHAS (see Supplementary Material 2 for course announcement).

This project was an educational evaluation that did not involve identifiable human subjects data, hence, no informed consent was obtained. According to the U.S. Department of Health and Human Services regulations (45 CFR 46), activities conducted only for educational evaluation purposes and not intended as research involving human subjects do not require IRB approval. Attendees were provided with the option of not completing the pre- and post-workshop evaluation surveys. Finally, their responses were anonymous.

### Evaluation

A pre- and post-workshop evaluation was designed using the Kirkpatrick Framework [24] to assess various aspects of the workshop’s impact: Level 1 (reaction), Level 2 (knowledge), and Level 3 (use). More specifically, the evaluation included questions on satisfaction and goal achievement (Level 1), changes in knowledge and skills (Level 2), and plans to apply the skills in current and future research (Level 3). The pre-workshop survey measured goals, experience, knowledge, and confidence in workshop topics, while the post-workshop survey measured change in knowledge and confidence and obtained feedback on the workshop. Reported knowledge and confidence questions included all the topics covered in the workshop and were responded to on a 4-point Likert scale (0: None, 1: A little, 2: Some, and 3: A lot). We chose a 4-point Likert scale to encourage clear responses by limiting the possibility of neutral or indecisive answers, which are more common in 5- or 7-point scales, an empirical finding which has been well-documented (e.g., see Garland 1991) [25]. The goal was to prompt respondents to lean more decisively toward either a positive or negative response. Moreover, we included open-ended questions to inform and improve future workshops and to gather any other feedback from the trainees. The time interval between the pre-survey and post-survey evaluations was three days. Pre- and post-evaluation surveys are provided in Supplementary Material 3. To ensure confidentiality and protect the identities of participants, a system of codes was employed during the evaluation process. This ensured that individual responses remained anonymous while still allowing matched analysis of the responses by individual. Finally, all attendees of the training participated in the evaluation survey, and no participants were excluded or declined to participate.

### Data analysis

All the analyses were conducted in IBM SPSS Statistics for Windows, Version 29.02 [26]. Demographic characteristics of the sample such as gender and educational level were examined using descriptive statistics. Paired samples t-tests were conducted to compare the pre- and post-workshop survey responses in knowledge and confidence in all topics covered in the workshop. Bonferroni corrections [27] were applied to adjust for multiple comparisons. The change in the rate of correct answers in assessing participants’ knowledge of PROMs, factor analysis, validity, and cognitive interviewing was investigated by the paired-samples proportions tests. Finally, open-ended questions were reviewed and synthesized to examine the qualitative feedback from the participants.

## Results

### Participants

A total of 27 individuals (44.4% male) attended the training. Most participants (*N* = 12, 44.4%) were from MUHAS. The remaining institutions included other hospitals (e.g., Muhimbili National Hospital, Ocean Road Cancer Institute, Singida Regional Hospital and others), national and district institutions including the President’s Office Public Service Recruitment Secretariat, the NIMR, and Bagamoyo District Council, other universities including the University of Dodoma and the University of Dar es Salaam. Research areas included quality of life in multiple populations, perceptions of healthcare workers on medication use in sickle cell disease, depression in adolescents, medication adherence, childbirth experience, satisfaction with care, and self-management. Most participants had research roles (*N* = 23, 85.0%) at their institutions, and almost half had a clinical role (*N* = 33, 48.2%) and were pursuing further training in their careers (see Table 2). Fewer than half of the participants (*N* = 12, 44.4%) had more than one year of experience in research using PROMs, 59.3% reported having limited level of research experience (e.g., 50% of participants reported being involved in projects but never having led one), and 48.1% reported a limited publication experience (see Table 2). Experience in PROMs areas such as selection, development, and adaptation were also low (see Table 3).

**Table 2:** Participants in the Patient-Reported outcomes (PROs) workshop

Characteristic	*N* (%)
Gender	
Male	15 (55.6)
Female	12 (44.4)
^a^Role	
Researcher	23 (85.0)
Clinician	13 (48.2)
Program Manager	5 (18.5)
PhD Candidate	11 (40.7)
Other	5 (18.5)
Highest education level completed	
University	11 (40.7)
Doctoral	16 (59.3)
^b^PCOR Experience	
Less than one year	15 (55.6)
More than one year	12 (44.4)
Research experience	
None	3(11.1)
Worked on projects but never led one	13(48.1)
Led 1 or 2 projects	4 (15.4)
Led more than 2 projects	7 (26.9)
Publication experience	
None	6 (22.2)
Yes, as a co-author	7 (25.9)
Yes, as a first author	13 (48.1)
Yes, as a senior author	1 (3.7)

^a^Participants selected more than one role

^b^*PCOR* Patient-Centered Outcomes Research

**Table 3:** Workshop participants’ experience level in patient reported outcomes measures (PROMs) tasks at the start of the workshop

Task	Completed Pre-Survey *N* = 27		
	No Experience	Involved but not leading	Leading
PROMs Selection	9 (33.3)	16 (59.3)	2 (7.4)
PROMs Development	25 (92.6)	1 (3.7)	1 (3.7)
PROMs Adaptation	14 (51.9)	9 (33.3)	4 (14.8)
PROMs Cultural Adaptation	15 (55.6)	7 (25.9)	5 (18.5)
Testing for Validity	20 (74.1)	7 (25.9)	-
Testing for Factor Analysis	25 (92.6)	2 (7.4)	-
PROMs Manuscript Writing	23 (85.2)	3 (11.1)	1 (3.7)

### Evaluation results

#### Kirkpatrick level 1: reaction

In total, 92% of the participants reported that the workshop was successful “a lot” in meeting its goals, and the remaining 8% reported that it was “somewhat” successful. In terms of personal goals, most participants (87.5%) indicated that the workshop met their personal goals “a lot” while all other participants reported that it “somewhat” met their personal goals.

Open-ended responses from participants indicated a high level of engagement throughout the workshop, particularly during interactive and collaborative sessions. Many attendees emphasized the value of group-based activities and discussions. One participant noted, “I felt most engaged when we discussed the linguistic translation of tools and conducted cognitive interviews and debriefings in groups, as we documented on flipcharts and ensured that everyone understood the exercise.” Others echoed this sentiment, with one participant stating, “Most of the time, the class was engaging; the materials provided were sufficient, and the facilitation was excellent!”. Another attendee highlighted the diversity of topics covered, sharing, “There were always different aspects of the same subject with new experiences.” while another one emphasized the benefits of peer learning, stating, “During group work and assignments because I got a chance to gain concepts from others’ perspectives.”

In contrast, lower engagement was reported during sessions that were more conceptually dense or scheduled toward the end of the workshop, when participants felt fatigued. For instance, one participant reflected, *“Using EFA in practice because it was my first time and most of the stuff was new*,*”* indicating that unfamiliar content posed a challenge. Another explained, *“During the session on sample size estimation*,* because I was tired*,*”* and a third elaborated on the difficulty of a later session: *“During the component analysis section. I think it was too much detail in a short time*,* and I was tired (last day).”* These reflections suggest that, while overall engagement was high, the timing and complexity of specific topics influenced participant experience.

#### Kirkpatrick level 2: knowledge

**Fig. 1:**
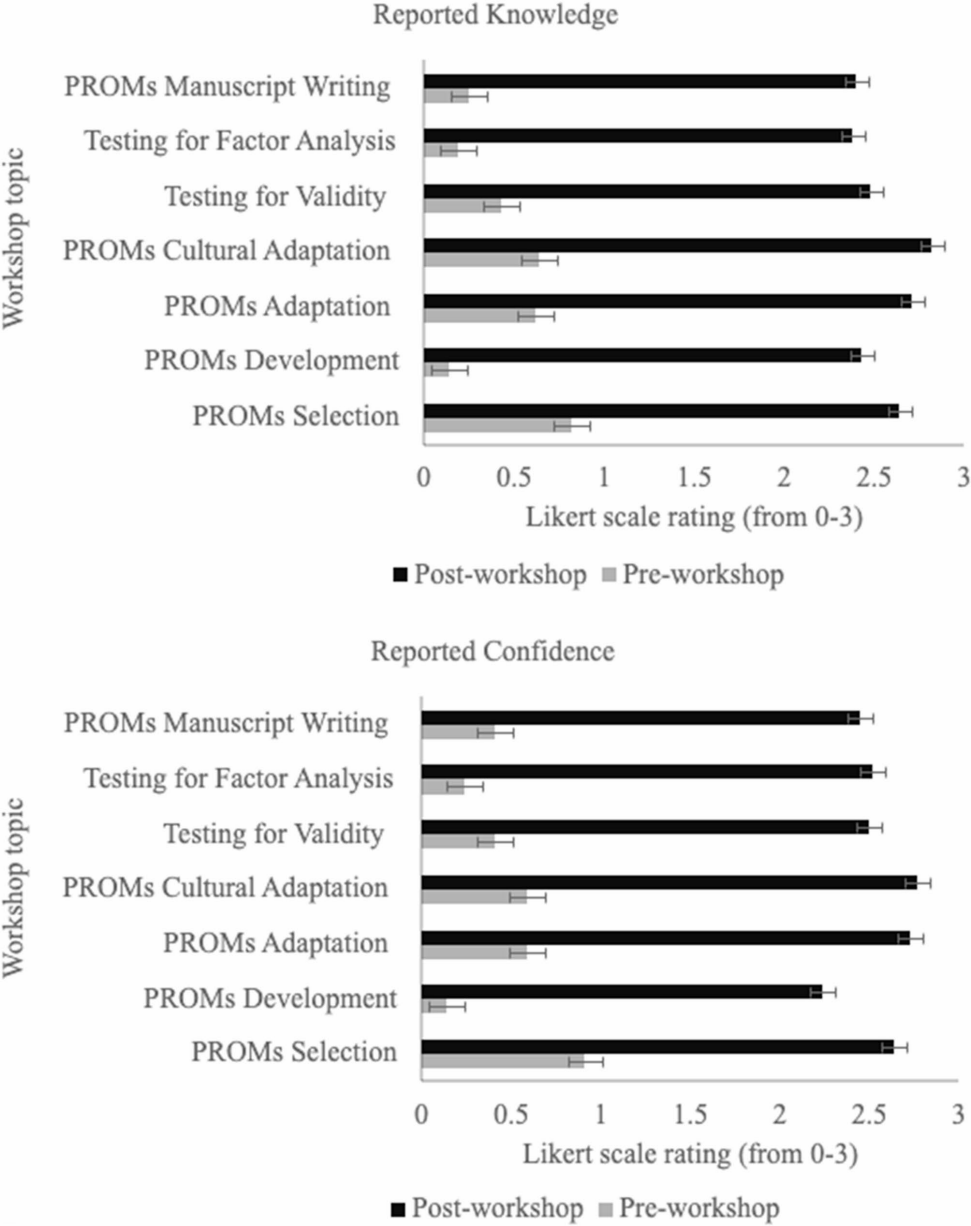
Pre- and post-workshop evaluations of reported knowledge and confidence

Participants displayed a significant increase both in their reported knowledge and confidence on all topics following the training workshop (Fig. 1). For all workshop topics, the increase in reported knowledge and confidence was significant (*p* <.001) (see Table S1 in the Supplementary Material 4). On average, reported knowledge for all topics increased from 0.44 (SD = 0.68) to 2.55 (SD = 0.56) while confidence rose from 0.47 (SD = 0.72) to 2.55 (SD = 0.56).

**Fig. 2:**
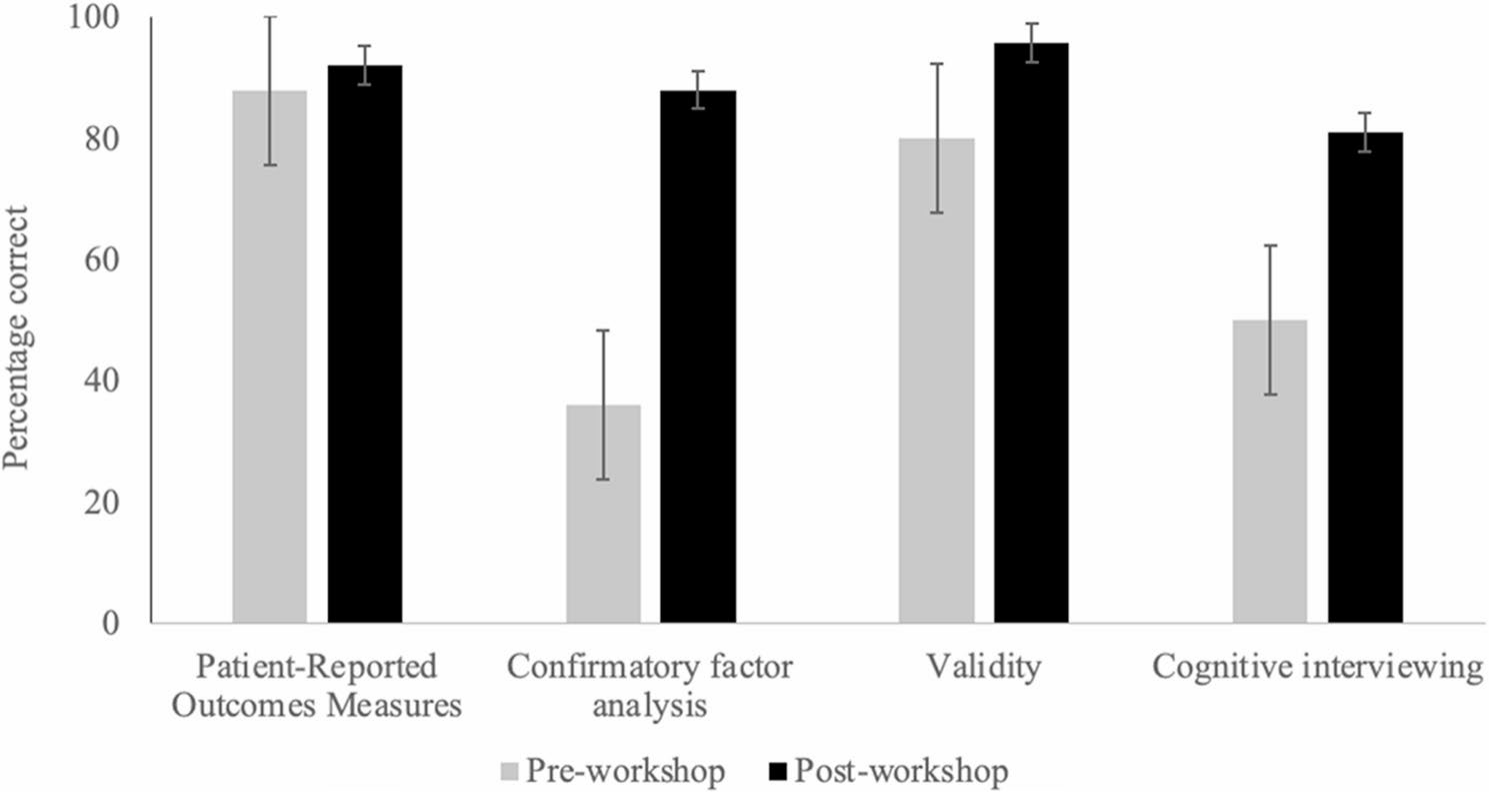
Percentage of correct answers on pre- and post-workshop assessment questions

Changes in the rates of correct answers assessing knowledge of PROMs, confirmatory factor analysis, validity, and cognitive interviewing displayed an increase for all questions, with the highest increase in the confirmatory factor analysis question, followed by cognitive interviewing, validity, and PROMs questions (Fig. 2). These observed differences were significant for the use of confirmatory factor analysis (*z* = −3.46, *p* <.001) and the cognitive interviewing (*z* = −1.67, *p* =.048) questions but not for the steps in using PROMs (*z* = −0.45, *p* =.327) and the divergent validity (*z* = −1.34, *p* =.090) questions. More specifically, participants had significantly more correct answers for using confirmatory factor analysis (88%) and cognitive interviewing (81%) compared to their pre-workshop performance (36% and 50%, respectively). They also had more correct answers for the steps in using PROMs (92%) and the divergent validity (95.8%) but this increase in the rate of their correct answers did not reach significance compared to the percentage of correct answers in their pre-workshop evaluations (88% and 80%, respectively).

#### Kirkpatrick level 3: use

All participants (100%) indicated their intention to apply the knowledge gained from the workshop to their current and future research, as reflected in their responses to the open-ended question: “Please explain how you will incorporate the workshop knowledge into your current research.” Their responses revealed several keyways in which they planned to do so, ranging from incorporating workshop content into research design to adapting and validating psychological measures within their local contexts.

Several participants expressed intentions to integrate the learning directly into their research design and measurement development. One participant noted, *“I am a PhD student*,* and I will incorporate the knowledge in developing my research tools*,*”* while another shared plans to assess their current instruments more rigorously: *“I will assess the tools I am using to do exploratory and confirmatory to understand the measurement instrument.”* Similarly, another participant stated, *“I plan to conduct a validity and reliability study of the quality of care for the people living with HIV to see if the measures in each construct of the tool could give the expected results.”*

Other attendees highlighted how the workshop had enhanced their capacity to participate in the validation and adaptation of psychological scales. As one participant put it, *“This learning will help me to participate in validating psychological scales in an implementation setting*,*”* while another stated, *“Most of the psychological scales are not validated in our setting. This learning helps me to participate in validating psychological scales which are implementation setting.”*

Finally, participants also noted specific applications to their projects. One reflected, *“My current project improves measurement of depression and anxiety*,* so definitely I am going to incorporate my learning.”* These responses suggest strong potential for the workshop to influence both individual research practices and broader measurement approaches in implementation science.

## Discussion

Northwestern University and MUHAS successfully collaborated to design and implement a workshop that effectively increased knowledge and confidence in research methods to support the adaptation and development of PROMs for use in Tanzania. Both quantitative and qualitative findings showed significant gains in participants’ knowledge and confidence in key areas of PROMs development and validation, with all attendees reporting plans to apply the skills in future research. The workshop also demonstrated the feasibility of building capacity in PROMs, including psychometric validation and future plans for integrating similar training into research and clinical care. By focusing on both the theoretical and practical aspects of developing, adapting, and validating PROMs, the workshop proved to be a successful model for advancing PCOR capabilities in LMICs.

Our findings echo broader calls for a growing focus on the need to effectively adapt tools validated in higher income settings to regions that are more under resourced such as in the Global South and Africa, as well as effectively develop new tools for people-reported outcomes in these settings [28]. As Laher (2024) notes in her review of psychological assessment in South Africa, Western-developed tools often dominate and are frequently imported without sufficient adaptation, leading to ongoing challenges with accessibility, cultural relevance, and equitable implementation [29]. Empirical evidence further supports this need: Rodriguez et al. (2025) evaluated the psychometric properties of the PHQ-9, GAD-7, and PC-PTSD-5 scales among South African adolescent girls and young women [30]. The GAD-7 displayed strong psychometric properties across linguistic groups, whereas the PHQ-9 and PC-PTSD-5 measures showed variability, indicating the need for ongoing adaptation and validation. Moreover, a systematic review by Wang et al. (2022), identified 88 HIV-specific PROMs, most of which lacked robust psychometric validation, with only three receiving a Class A rating based on the COSMIN (COnsensus-based Standards for the selection of health status Measurement INstruments) checklist [31, 32]. Together, these findings reinforce the importance of our work in building research capacity among Tanzanian researchers and clinicians to adapt, develop, and validate PROMs that are not only scientifically sound but also culturally responsive.

This initiative aligns with global efforts to achieve the UNAIDS 95-95-95 targets by addressing gaps in healthcare delivery and outcomes. Workshops designed to build research capacity in adapting and validating PROMs are vital as a first step toward equipping local researchers to generate and evaluate tools that can inform more people-centered health system interventions designed to increase people-centeredness of care to better address HIV as a chronic illness. Locally, the capacity built in the workshop will supported Tanzania’s broader healthcare goals of improving quality of life and addressing health inequities among PLWH through adaptation and development of culturally relevant and locally validated PROMs in the region.

With one exception, we were not able to find other comparable studies on workshops to increase the capacity for psychometrics in PROMs in similar contexts. A similar effort in Rwanda integrated PROMs training into courses for pediatric physiotherapists [33]. They demonstrated that structured training improved participants’ knowledge and confidence in using PROMs for patient-centered care. Both our workshop in Tanzania and the training program in Rwanda combined theoretical teaching with hands-on practical components. They emphasized sustainability through buy-in from local leadership (e.g., MUHAS institutional leadership championing the workshop). The relative lack of studies in the literature underscores the need for further effective capacity-building training efforts to adapt and develop PROMs for use in clinical populations in African countries.

Our study has several strengths. First, it was grounded in an international collaboration between Northwestern University and MUHAS, combining Northwestern’s longstanding expertise in patient-reported outcome measure development and validation with MUHAS’s deep contextual knowledge and understanding of the Tanzanian health system and population. Second, using the Kirkpatrick Framework enabled a structured, theory-based evaluation across multiple levels of impact. Third, employing both quantitative and qualitative methods provided a comprehensive understanding of participant learning and satisfaction. Finally, the focus on PROMs development and validation addressed a critical training gap in a low-resource setting, and participants’ reported plans for real-world application highlight the workshop’s practical value.

This study is not without its limitations. Reliance on self-reported measures of knowledge and confidence, while useful, may only partially capture the long-term application of skills. Another limitation of this study is that workshop participation required at least a master’s degree, primarily in health-related fields, which may limit the applicability of the training and PROM use among broader clinical staff. Future efforts should consider more inclusive training approaches to support wider implementation. Additionally, the participant feedback was overwhelmingly positive, but the absence of a control group suggests a future opportunity to assess the workshop’s effects more clearly. Future workshops should focus on expanding participant diversity and incorporating longitudinal follow-up to better evaluate the application of learned skills in both clinical and research settings.

Finally, the policy recommendations emerging from our work include supporting the integration of PROMs training into existing national research capacity-building initiatives and continuing medical education. Such investments would strengthen health systems’ ability to monitor not only clinical outcomes but also patient-reported experiences and quality of life—critical domains in chronic disease care, including HIV. As countries work toward achieving the UNAIDS 95-95-95 targets and broader health equity goals, embedding PROMs well-validated to global contexts into clinical care and health system evaluation will be vital.

## Conclusions

The workshop enhanced the capacity and commitment of participants to adapt and validate, PROMs in Tanzania. Plans are in place to develop a certificate course in Psychometrics led by faculty and trainees from MUHAS. Additionally, the feasibility and value of scaling similar training workshops will be assessed to strengthen HIV PCOR capacity in other countries within the region.

In conclusion, our findings highlight the role of targeted training programs in building capacity for PCOR in LMICs. By equipping researchers, clinicians, and policymakers with the skills to develop, adapt, and validate PROMs, such initiatives can enhance the integration of patient-centered approaches into health systems. In particular, engaging clinicians and policymakers is critical to translating PROMs into practice and policy, thereby improving healthcare outcomes and quality of life for PLWH and other populations. Expanding these efforts through sustained investment and collaboration across global and local stakeholders will be essential for addressing the complex healthcare challenges facing LMICs.

## Supplementary Information

Supplementary Material 1.

Supplementary Material 2.

Supplementary Material 3.

Supplementary Material 4.

## Data Availability

The datasets and materials used and/or analyzed during the current study are available from the corresponding author on reasonable request.

## Abbreviations

PLWH: People Living with HIV
PCOR: Patient-Centered Outcomes Research
PROs: Patient-Reported Outcomes
PROMs: Patient-Reported Outcome Measures
LMICs: Low- and Middle-Income Countries
MUHAS: Muhimbili University of Health and Allied Sciences
NIMR: National Institute of Medical Research

## References

[1] Hiliza J, Ndizeimana E, William H, Lebba J, Musanhu C, Nsubuga P et al. Accelerating HIV and AIDS services delivery in Kigoma region, Tanzania. Pan Afr Med J. 2023;45(1):9. 10.11604/pamj.supp.2023.45.1.39597 PMC10395105 37538364

[2] Kahabuka MS, Woldeamanuel Y, Mbelele PM, Mpolya EA, Mpagama SG, Kessy JP, et al. HIV viral suppression and risk of viral rebound in patients on antiretroviral therapy: a two- year retrospective cohort study in Northern Tanzania. BMC Infect Dis. 2024;24:390. 38605325 10.1186/s12879-024-09161-y PMC11007878

[3] World Health Organization. Consolidated guidelines on the use of antiretroviral drugs for treating and preventing HIV infection: recommendations for a public health approach. 2nd ed. Geneva: World Health Organization; 2016. 27466667

[4] Beres LK, Underwood A, Le Tourneau N, Kemp CG, Kore G, Yaeger L, et al. Person-centred interventions to improve patient– provider relationships for HIV services in low‐ and middle‐income countries: a systematic review. J Int AIDS Soc. 2024;27:e26258. 38740547 10.1002/jia2.26258 PMC11090778

[5] Bristowe K, Clift P, James R, Josh J, Platt M, Whetham J, et al. Towards person-centred care for people living with HIV: what core outcomes matter, and how might we assess them? A cross‐national multi‐centre qualitative study with key stakeholders. HIV Med. 2019;20:542–54. 31162817 10.1111/hiv.12758

[6] Lazarus JV, Safreed-Harmon K, Barton SE, Costagliola D, Dedes N, Del Amo Valero J, et al. Beyond viral suppression of HIV– the new quality of life frontier. BMC Med. 2016;14:94. s12916-016-0640–4. 27334606 10.1186/s12916-016-0640-4 PMC4916540

[7] Egger JR, Kaaya S, Swai P, Lawala P, Ndelwa L, Temu J, et al. Functioning and quality of life among treatment-engaged adults with psychotic disorders in urban tanzania: baseline results from the KUPAA clinical trial. PLoS ONE. 2024;19:e0304367. 38889160 10.1371/journal.pone.0304367 PMC11185462

[8] Saronga HP, Kaaya S, Fawzi MCS. Cost-effectiveness of the healthy options group psychosocial intervention for perinatal women living with HIV and depression in Tanzania. PLOS Ment Health. 2024;1:e0000066. 10.1371/journal.pmen.0000066 PMC12798287 41661746

[9] Aantjes CJ, Quinlan TK, Bunders JF. Practicalities and challenges in re-orienting the health system in Zambia for treating chronic conditions. BMC Health Serv Res. 2014;14:295. 25005125 10.1186/1472-6963-14-295 PMC4094789

[10] Malapati SH, Edelen MO, Nthumba PM, Ranganathan K, Pusic AL. Barriers to the use of Patient-Reported outcome measures in Low- and Middle-income countries. Plast Reconstr Surg - Global Open. 2024;12:e5576. 10.1097/GOX.0000000000005576 PMC10843469 38317651

[11] McKown S, Acquadro C, Anfray C, Arnold B, Eremenco S, Giroudet C, et al. Good practices for the translation, cultural adaptation, and linguistic validation of clinician-reported outcome, observer-reported outcome, and performance outcome measures. J Patient Rep Outcomes. 2020;4:89. 33146755 10.1186/s41687-020-00248-z PMC7642163

[12] Patrick DL, Burke LB, Gwaltney CJ, Leidy NK, Martin ML, Molsen E, et al. Content Validity—Establishing and reporting the evidence in newly developed Patient-Reported outcomes (PRO) instruments for medical product evaluation: ISPOR PRO good research practices task force report: part 2—Assessing respondent Understanding. Value Health. 2011;14:978–88. 22152166 10.1016/j.jval.2011.06.013

[13] Prakash V, Shah S, Hariohm K. Cross-cultural adaptation of patient-reported outcome measures: A solution or a problem? Annals Phys Rehabilitation Med. 2019;62:174–7. 10.1016/j.rehab.2019.01.006 30753895

[14] Croome N, Ahluwalia M, Hughes LD, Abas M. Patient-reported barriers and facilitators to antiretroviral adherence in sub-Saharan Africa. AIDS. 2017;31:995–1007. 28121707 10.1097/QAD.0000000000001416 PMC5378008

[15] Cipta DA, Andoko D, Theja A, Utama AVE, Hendrik H, William DG, et al. Culturally sensitive patient-centered healthcare: a focus on health behavior modification in low and middle-income nations—insights from Indonesia. Front Med. 2024;11:1353037. 10.3389/fmed.2024.1353037 PMC11047771 38681051

[16] Goggin K, Gqaleni N, Mbhele AL, Makhathini ME, Buthelezi TD, Ndlovu SW, et al. The translation and cultural adaptation of Patient-reported outcome measures for a clinical study involving traditional health providers and Bio-medically trained practitioners. Alternation (Durb). 2010;17:273–94. 25309104 PMC4191735

[17] Ngwayi JRM, Obie KU, Tan J, Xu J, Alizada M, Porter DE. Chinese cross-culturally adapted patient-reported outcome measures (PROMs) for knee disorders: a systematic review and assessment using the evaluating the measurement of Patient-Reported outcomes (EMPRO) instrument. J Orthop Surg Res. 2022;17:508. 36434665 10.1186/s13018-022-03399-5 PMC9694593

[18] van Ommeren M, Sharma B, Thapa S, Makaju R, Prasain D, Bhattarai R, et al. Preparing instruments for transcultural research: use of the translation monitoring form with Nepali-Speaking Bhutanese refugees. Transcult Psychiatry. 1999;36:285–301.

[19] Borsa JC, Damásio BF, Bandeira DR. Cross-cultural adaptation and validation of psychological instruments: some considerations. Paidéia (Ribeirão Preto). 2012;22:423–32.

[20] Fogarty International Center. HIV research training program in tanzania: enhancing capacity for patient-centered outcomes research in HIV care and prevention. Natl Institutes Health. 2023. Accessed 15 Oct 2024.

[21] Osati E, Fred, Shayo G, Ambrose, Sangeda R, Zozimus. Rugemalila, joan. Patient-Reported health outcomes among HIV-Infected patients on antiretroviral therapy in a tertiary hospital in Dar Es salaam, tanzania: A cross sectional study. J AIDS HIV Treat. 2020;2(1):1–11.

[22] Zachariah R, Ford N, Philips M, Massaquoi SL, Janssens M. Task shifting in HIV/AIDS: opportunities, challenges and proposed actions for sub-Saharan Africa. Trans R Soc Trop Med Hyg. 2009;103:549–58. 18992905 10.1016/j.trstmh.2008.09.019

[23] Oronje RN, Mukiira C, Kahurani E, Murunga V. Training and mentorship as a tool for Building African researchers’ capacity in knowledge translation. PLoS ONE. 2022;17:e0266106. 35358255 10.1371/journal.pone.0266106 PMC8970368

[24] Kirkpatrick DL. Evaluating training programs: the four levels. 1. paperback print. San Francisco: Berrett-Koehler; 1996.

[25] Garland R. The Mid-Point on a Rating Scale: Is it Desirable? Mark Bull. 1991;2(1):66–70.

[26] IBM Corp. IBM SPSS statistics for windows, version 29.0.2.0, Armonk. NY: IBM Corp; 2023.

[27] Dunn OJ. Multiple comparisons among means. J Am Stat Assoc. 1961;56:52–64.

[28] Van Zyl C, Mokkink LB, Derman W, Hanekom S, Heine M. Patient-Reported outcome measures in key Sub-Saharan African languages to promote diversity: A scoping review. Value Health Reg Issues. 2023;34:86–99. 36621214 10.1016/j.vhri.2022.11.001

[29] Laher S. Advancing an agenda for psychological assessment in South Africa. South Afr J Psychol. 2024;54:515–30. 10.4102/ajopa.v6i0.166 PMC12082265 40406656

[30] Rodriguez VJ, Emerson E, Atujuna M, Ngcuka A, Jaworski E, Macdonald P, et al. Psychometric properties of mental health screening tools in South African adolescent girls and young women. J Affect Disord. 2025;377:148–56. 39983777 10.1016/j.jad.2025.02.043

[31] Wang Z, Zhu Y, Duan X, Kang H, Qu B. HIV-Specific reported outcome measures: systematic review of psychometric properties. JMIR Public Health Surveill. 2022;8:e39015. 36222289 10.2196/39015 PMC9782451

[32] Mokkink LB, Terwee CB, Patrick DL, Alonso J, Stratford PW, Knol DL, et al. The COSMIN checklist for assessing the methodological quality of studies on measurement properties of health status measurement instruments: an international Delphi study. Qual Life Res. 2010;19:539–49. 20169472 10.1007/s11136-010-9606-8 PMC2852520

[33] Mann M, Musabyemariya I, Harding L, Braxley B. Using Patient-Reported outcome measures to promote Patient-Centered practice: Building capacity among pediatric physiotherapists in Rwanda. Glob Health Sci Pract. 2020;8:596–605. 33008866 10.9745/GHSP-D-19-00408 PMC7541114

