# Supplementary material for: Building research capacity to adapt and develop Patient-Reported outcome measures in low- and middle-income countries: results from a psychometrics workshop in Tanzania
Source: BMC Health Serv Res. 2025 Jul 1;25:871. doi: 10.1186/s12913-025-13064-2 (PMC12219237; doi:10.1186/s12913-025-13064-2)
Supplement: Supplementary file 1 — Supplementary Material 1. [file 12913_2025_13064_MOESM1_ESM.docx]

**Supplementary Material 1**

**Agenda**

**Introduction to Psychometrics Workshop**

**Muhimbili University of Health and Allied Sciences (MUHAS) in collaboration with the Feinberg School of Medicine, Northwestern University**

***‘Building capacity for patient centered outcomes research to improve the quality and impact of HIV Care in Tanzania’ NIH Fogarty 1D43TW010946***

**29 Jan- 1 Feb 2024, Dar es Salaam, Tanzania**

**TRAINING OBJECTIVES:**

1. **Understanding Assessment Instruments:**
   - To understand how assessment instruments are developed and used.
   - To identify the questions that need to be answered before an instrument is deemed ready for use in a new context.
2. **Instrument Selection and Evaluation:**
   - To demonstrate the process of selecting standardized assessment instruments.
   - To evaluate the strengths and weaknesses of selected instruments.
3. **Linguistic and Cultural Adaptation:**

- To teach attendees how to perform linguistic and cultural adaptation for tools validated in other settings, languages, or populations.

1. **Psychometric and Assessment Principles:**
   - To demonstrate the analysis of basic psychometric and assessment principles.
   - To apply these principles to adaptations of research assessment instruments measuring psychosocial attributes or other health-relevant outcomes.
   - To interpret results of psychometric analyses or testing.
2. **Understanding Published Research:**
   - To develop trainees' understanding of published research using assessment instruments.
3. **Ethical and Multicultural Awareness:**
   - To increase trainees' understanding of the ethical issues surrounding patient-reported outcomes and other validated survey questionnaires.
   - To increase awareness of multicultural issues surrounding psychological-health measures.

**LEARNING OUTCOMES:**

1. **Psychometric Evaluation:**
   - Critically evaluate psychological and health measures based on their psychometric merits.
2. **Adaptation Planning:**
   - Plan for linguistic and cultural adaptation of validated tools for use in new settings and populations.
3. **Research Skills:**
   - Evaluate current research on psychological and health assessment instruments related to trainee’s own planned research/clinical assessment interests.
4. **Psychometric Knowledge:**
   - Have a working knowledge of psychometric theory and the psychometric qualities of tests.
   - Interpret results and collaborate to plan future analysis.
5. **Ethical and Multicultural Appreciation:**
   - Appreciate and understand ethical issues surrounding psychological and health measures.

**LECTURERS:**

- **Northwestern University (NU):**
- Emily Ho, PhD
- Berivan Ece, PhD
- Lisa Hirschhorn, MD MPH
- **Muhimbili University of Health and Allied Sciences (MUHAS):**
- Candida Moshiro, PhD
- Theresia Ottaru, PhD
- Linda Mlunde, PhD
- Mrema Kilonzo, PhD
- Sylvia Kaaya, MD, PhD

**SMALL GROUP FACILITATORS (approximately 4 groups):**

- Linda Mlunde
- Veneranda Bwana
- Innocent Yusufu
- Mrema Kilonzo
- Sylvia Kaaya
- Candida Moshiro
- Alphoncina Kagaigai
- Helen Siril
- Claudia Hawkins
- Berivan Ece
- Emily Ho
- Lisa Hirschhorn

**MATERIALS**

**Pre-reads:**

- Mlunde, L. B., Hirschhorn, L. R., Nyblade, L., Rothrock, N. E., Mbugi, E. V., Moskowitz, J. T., Kaaya, S., Hawkins, C., Leyna, G., & Mbwambo, J. K. (2023). Translation and cultural adaptation of drug use stigma and HIV stigma measures among people who use drugs in Tanzania. *PloS one, 18*(10), e0292642. <https://doi.org/10.1371/journal.pone.0292642>
- Kagaigai, A., Anaeli, A., Mori, A. T., & Grepperud, S. (2021). Do household perceptions influence enrollment decisions into community-based health insurance schemes in Tanzania?. *BMC health services research, 21*(1), 162. <https://doi.org/10.1186/s12913-021-06167-z>
- Ho, E. H., Hagmann, D., & Loewenstein, G. (2020). Measuring Information Preferences. *Management Science*, mnsc.2019.3543. <https://doi.org/10.1287/mnsc.2019.3543>
- Ece, B., Aytürk, E., Göktaş, N., et al. (2023). Factorial structure of autobiographical recollection assessed by a Turkish version of Autobiographical Recollection Test (ART). *Current Psychology, 42*, 10894–10909. <https://doi.org/10.1007/s12144-021-02388-x>
- Maman, S., Mulawa, M. I., Balvanz, P., McNaughton Reyes, H. L., Kilonzo, M. N., Yamanis, T. J., Singh, B., & Kajula, L. J. (2020). Results from a cluster-randomized trial to evaluate a microfinance and peer health leadership intervention to prevent HIV and intimate partner violence among social networks of Tanzanian men. *PLOS ONE*, *15*(3), e0230371. <https://doi.org/10.1371/journal.pone.0230371>
- Tse, E. T. Y., Lam, C. L. K., Wong, C. K. H., Chin, W. Y., Etz, R. S., Zyzanski, S. J., & Stange, K. C. (2020). Cultural adaptation and content validity of a Chinese translation of the ‘Person-Centered Primary Care Measure’: Findings from cognitive debriefing. *Family Medicine and Community Health*, *8*(4), e000621. <https://doi.org/10.1136/fmch-2020-000621>

**TIMETABLE**

| **TIME** | **TOPIC** | **PRESENTER** |
| --- | --- | --- |
| **DAY ONE**: **Jan 30, Tuesday**  **FOUNDATIONS of PSYCHOMETRICS** | | |
| 8:30 - 9:00 | **Registration and pre-work Evaluation** |  |
| 9:00 - 9:15 | **Welcoming remarks**  **Overview of the PCOR D43**  **Introduction of participants** | Erasto  Sylvia Kaaya  Lisa Hirschhorn  Claudia Hawkins |
| 9:15 - 9:45 | **Introduction and Brief History of Psychometrics**   - **How are Measures Created?** - Scale creation - Mapping theory to items | Presentation:  Emily Ho  Berivan Ece |
| 9:45 - 10:30 | **Tell us about your measurement tool**   - Has it been used in your setting? - What is the validity evidence? - How was it adapted? - Psychometric evidence (e.g., EFA/CFA)? - Where has it been used? | Moderator:  Sylvia Kaaya  Assisted by  Lisa Hirschhorn  Hellen Siril |
| 10:30 -11:00 | **MORNING BREAK** |  |
| 11:00 –11:25 | **Why Adapt Existing Measures? Measure Translation, Cultural Adaptation, and Validation of the Cultural Adaptation (Principles and Methods)**   - Tool selection - Linguistic translation and semantic equivalence - Face validity | Presentation:  Emily Ho  Berivan Ece |
| 11:25-12:00 | **Small group discussion on example of linguistic translations** | Presentation:  Linda: Mlunde 5 minutes –report from the field  Small group work  Brief report back (1-2 groups) |
| 12-12:35 | **Measure Translation, Cultural Adaptation, and Validation of the Cultural Adaptation (Principles and Methods; continued)**   - Cultural adaptation and implications - Cognitive debriefing | Linda Mlunde |
| 12:35-13:00 | **Small group exercise:**  Cognitive debrief – role-play | Introduction to exercise: Sylvia Kaaya and Lisa Hirschhorn  Facilitators:  Sylia Kaaya, Linda Mlunde, Hellen Siril, team |
| 13:00 -14:00 | **LUNCH BREAK** |  |
| 14:00 -14:35 | **Measure Translation, Cultural Adaptation, and Validation of the Cultural Adaptation (Principles and Methods and experience**   - Tool adaptation - Pilot testing | Claudia Hawkins moderator    **Examples from the field**:  Veneranda Bwana (5 minutes)  Innocent Yusufu (5 minutes)  Hellen Siril (5 minutes) |
| 14:35-15:15 | **Small group exercise:**  Tool adaptation and pilot testing exercise | Introduction to exercise: Sylvia Kaaya |
| 15:15-15:30 | **Small group presentation** | Moderator: Emily Ho |
| 15:30 -16:00 | **Reflections and insights from attendees** | *Moderator*:  Sylvia Kaaya |
| **DAY TWO: Jan 31, Wednesday**  **PRACTICAL APPLICATION and ADAPTATION** | | |
| 9:00 - 9:25 | **Recap** | Attendees  Moderator: Lisa Hirschhorn |
| 9:25 -10:00 | **Psychometric Properties of a Measure: Reliability of a Test** | Presentation:  Emily Ho  Berivan Ece |
| 10:00-10:30 | **Reliability of a Test:**   - Output guided interpretation - Interpretation exercise (Small groups) | Emily Ho  Berivan Ece |
| 10:30 -11:00 | **MORNING BREAK** |  |
| 11:00 -11:40 | **Psychometric Properties of a Measure: Validity of a Test** | Presentation:  Emily Ho  Berivan Ece |
| 11:40 - 12:10 | **Validity of a Test:**   - Output guided interpretation - Interpretation exercise (Small groups) | Emily Ho  Berivan Ece |
| 12:10 - 13:00 | **Validity and Reliability: Interpreting Results of a Test** (group exercise) | Introduction of the exercise:  **Candida Moshiro** |
| 13:00 -13:30 | **Small group presentation** | Facilitator: Candida Moshiro |
| 13:30 -14:30 | **LUNCH BREAK** |  |
| 14:30 –15:00 | **Real world example: Working with results from a study** | Mrema Kilonzo |
| 15:00-15:45 | **Office hours on current research projects** | Mrema Kilonzo  Emily Ho  Berivan Ece  Sylvia Kaaya |
| 15:45-16:00 | **Reflections from attendees** | Lisa Hirschhorn and Mrema Kilonzo facilitate  Attendees |
| **DAY THREE: Feb 1, Thursday**  **STATISTICAL ANALYSIS, MORE PRACTICAL ACTIVITIES AND ETHICAL CONSIDERATIONS** | | |
| 9:00 - 9:30 | **Recap** | Attendees  Moderator: Claudia Hawkins |
| 9:30 -10:00 | **Introduction to Factor Analysis (PCA, EFA, CFA):** When to use which? | Presentation:  Emily Ho  Berivan Ece |
| 10:00 - 10:30 | **Output guided interpretation** | Berivan Ece |
| 10:30 -11:00 | **MORNING BREAK** |  |
| 11:00-11:15 | **Practical exercise in data interpretation and experience from a study** | Alphoncina Kagaigai |
| 11:15 -13:00 | **Demonstration of EFA and CFA with data**    **Using EFA with data to interpret and evaluate psychometric properties of a test** | Introduce exercise: Berivan Ece  Facilitators: Alphoncina Kagaigai  Berivan Ece  Emily Ho  Candida Moshiro |
| 13:00 -13:30 | **Small group presentation** | Facilitator: Emily Ho |
| 13:30 -14:30 | **LUNCH BREAK** | **Office hours with Emily, Berivan, and Candida and Mrema** |
| 14:30 - 15:00 | **Real-world considerations for testing your adapted measure (e.g., guidelines, sample size)** | Candida Moshiro |
| 15:00 – 15:20 | **Publishing your results:** | Presentation:  Emily Ho  Berivan Ece |
| 15:20-15:35 | **Ethical considerations in doing psychometric work** | Sylvia Kaaya |
| 15:35- 16:00 | **Evaluation and Worksheet on next steps** | Moderators:  Lisa Hirschhorn  Claudia Hawkins |
| 16:00-16:30 | **Evaluation and Certificates!** | Workshop leads |
