# Supplementary material for: Building research capacity to adapt and develop Patient-Reported outcome measures in low- and middle-income countries: results from a psychometrics workshop in Tanzania
Source: BMC Health Serv Res. 2025 Jul 1;25:871. doi: 10.1186/s12913-025-13064-2 (PMC12219237; doi:10.1186/s12913-025-13064-2)
Supplement: Supplementary file 2 — Supplementary Material 2. [file 12913_2025_13064_MOESM2_ESM.docx]

**Supplementary Material 2**

**PATIENT-CENTERED OUTCOMES RESEARCH (PCOR) PROJECT**

**Training workshop announcement**

**Introduction to Psychometrics**

Muhimbili University of Health and Allied Sciences (MUHAS) in collaboration with Northwestern University (NU) will conduct a three-day workshop on Introduction to Psychometrics. This workshop aims to provide academic staff, postgraduate students, and other researchers with the skills needed to choose, adapt, and validate patient reported outcome measurement (PROM) tools. Participants will learn about the characteristics and principles of developing assessment instruments, selection of standardized measurement, how to linguistically and culturally adapt PROMS, and test validity in new settings and populations as well, and ethical and multicultural issues in PROMs development and use. The workshop will be delivered by highly qualified and experienced experts in psychometrics from NU and MUHAS and will include in-person lectures, discussions, and individual and group activities. Participants who complete the three days and related work will be awarded certificates of completion.

**Learning Outcomes:**

At the end of the workshop, attendees will be able to:

1. Understand principles and methods in cultural adaptation and tool validation
2. Understand and test different psychometric properties of a tool
3. Interpret results of different types of reliability and validity of a tool
4. Understand and interpret the principles of factor analysis

**Eligibility:**

To be eligible to apply, applicants must have at least a master’s degree, and experience in or plans for using PROMs in research or clinical work

**Application procedures:**

Send an email including your name, position, current research project, and a short paragraph on what you expect to get out of the training. In addition, include commitment to attend for all 3 days and briefly describe your experience with using PROMS.

Mr. Sebe Mohamed [sebejr@yahoo.com;](mailto:sebejr@yahoo.com) Mobile: +255 715 209 320

Last date for receiving applications is **5.00pm on 16^th^ January 2024**

**Course duration and venue**

**January 29^th^ – February 1, 2024**

**8:00 AM - 4:00 PM**

**MUHAS-CHPE building, 2^nd^ Floor Seminar Room**

**Course fee:** the organizers will cover the course fee, venue, health break refreshments, and lunch

For any further queries please contact Prof. Sylvia Kaaya [skaaya@gmail.com](mailto:skaaya@gmail.com) or Dr. Theresia Ambrose: [tambroce@yahoo.com](mailto:tambroce@yahoo.com)
