# Supplementary material for: Building research capacity to adapt and develop Patient-Reported outcome measures in low- and middle-income countries: results from a psychometrics workshop in Tanzania
Source: BMC Health Serv Res. 2025 Jul 1;25:871. doi: 10.1186/s12913-025-13064-2 (PMC12219237; doi:10.1186/s12913-025-13064-2)
Supplement: Supplementary file 3 — Supplementary Material 3. [file 12913_2025_13064_MOESM3_ESM.docx]

**Supplementary Material 3**

**PRE-EVALUATION**
**Introduction to Psychometrics Workshop**

**Muhimbili University of Health and Allied Sciences (MUHAS) in collaboration**

**with the Feinberg School of Medicine, Northwestern University**

***‘Building capacity for patient centered outcomes research to improve the quality and impact of HIV Care in Tanzania’ NIH Fogarty 1D43TW010946***

**29 Jan- 1 Feb 2024, Dar es Salaam, Tanzania**

We would like to understand a bit about who you are, your current knowledge and experience in the main areas of training. After the workshop, we will also ask you some questions and your feedback about the workshop to help us continue to strengthen the work and its responsiveness to your and others’ needs.

1. **First Three letters of your surname name + last 2 numbers of your year of birth:**
   1. **___ ____ ____ ___ __**
2. **What is your self-identified gender**

______ male

______ female

______ other (please describe _______________)

1. **What is the name of the institution where you currently work or are in school:** _____________________________
2. **What is your role(s) (Check all that apply)**

___researcher

___clinician

___program management

___student

___other

1. **What is the highest level of education you completed**

____Less than primary school

____Primary School

____Secondary School

____University

____Beyond University (ex. PhD, MD, etc)

1. **How long have you worked in PCOR (patient centered outcome research)?**

____I am new

____< 1 year

____1-5 years

____> 5 years

If you have done PCOR, please briefly list the last 1-2 projects

1. **How much research in any field have you done in the past?**

____ None

____I have worked on projects but never led a project

____I have led 1 or 2 projects

____I have led more than 2 projects

1. **Have you been involved in publishing a peer-reviewed research manuscript (check all that apply)?**

_____ No

_____ Yes, as a coauthor

_____ Yes as a first author

_____ Yes as a senior author

**9. Have you been involved in writing a research grant (check all that apply)?**

____ No

____ Yes as part of a team

____ Yes as the lead/principal investigator

10. **Please would you describe your experience in the following areas of research:**

|  | I have no experience | Involved but not leading | Leading |
| --- | --- | --- | --- |
| Choosing patient reported outcome measures (PROMS) |  |  |  |
| Developing a new PROM |  |  |  |
| Adapting PROMS in general |  |  |  |
| Cultural adaptation of PROMS |  |  |  |
| Testing for validity |  |  |  |
| Doing confirmatory or exploratory factor analysis (CFA or EFA) |  |  |  |
| Writing a manuscript describing the process of PROM development or adaptation? |  |  |  |
| Working on the manuscript revision process after journal submission |  |  |  |

**11. Please rate yourself in the following areas:**

| a. How would you rate your **knowledge** in the following areas of qualitative research: | | | | |
| --- | --- | --- | --- | --- |
|  | **None** | **A little** | **Some** | **A lot** |
| Choosing patient reported outcome measures (PROMS) |  |  |  |  |
| Developing a new PROM |  |  |  |  |
| Adapting PROMS in general |  |  |  |  |
| Cultural adaptation of PROMS |  |  |  |  |
| Testing for validity |  |  |  |  |
| Doing confirmatory or exploratory factor analysis (CFA or EFA) |  |  |  |  |
| Writing a manuscript describing the process of PROM development or adaptation? |  |  |  |  |
|  |  |  |  |  |
| B. How would you rate your **confidence** in the following areas of qualitative research: | | | | |
|  | **None** | **A little** | **Some** | **A lot** |
| Choosing patient reported outcome measures (PROMS) |  |  |  |  |
| Developing a new PROM |  |  |  |  |
| Adapting PROMS in general |  |  |  |  |
| Cultural adaptation of PROMS |  |  |  |  |
| Testing for validity |  |  |  |  |
| Doing confirmatory or exploratory factor analysis (CFA or EFA) |  |  |  |  |
| Writing a manuscript describing the process of PROM development or adaptation? |  |  |  |  |

For each question, choose the one correct answer.

**12**. In a new setting, steps needed for using an existing PROM include:

a) back and forward translations

b) assessing for semantic equivalence

c) cognitive interviewing

d) all of the above

e) none of the above

**13.** In developing or adapting a measure, Confirmatory Factor Analysis (CFA) should be used:

a) before PCA (principal components analysis) and EFA (exploratory factor analysis)
b) after PCA and before EFA

c) after PCA and EFA

d) the order does not matter

e) none of the above

**14.** If two measures exhibit divergent validity, that means:

a) the measures are highly correlated with each other

b) the measures are not very correlated with each other

c) one measure positively predicts the other measure at a future point in time

d) the measures represent all aspects of a given construct

e) none of the above

**15.** In adapting a measure, cognitive interviewing is a:

a) quantitative technique to assess reliability and validity of a measure

b) qualitative technique to assess expert opinions on semantic clarity of measure

c) quantitative technique to determine the underlying factor structure of a measure

d) qualitative technique to translate a measure from the source language to the target language

e) none of the above

**16.** **What are your 1-2 main goals of joining the workshop? (free text)**

1.

2.

**POST-EVALAUTION**
**Introduction to Psychometrics Workshop**

**Muhimbili University of Health and Allied Sciences (MUHAS) in collaboration with the Feinberg School of Medicine, Northwestern University**

***‘Building capacity for patient centered outcomes research to improve the quality and impact of HIV Care in Tanzania’ NIH Fogarty 1D43TW010946***

**29 Jan- 1 Feb 2024, Dar es Salaam, Tanzania**

We would like to understand a bit about who you are, your current knowledge and experience in the main areas of training. After the workshop, we will also ask you some questions and your feedback about the workshop to help us continue to strengthen the work and its responsiveness to your and others needs

1. **First Three letters of your surname name + last 2 numbers of your year of birth:**
   1. **___ ____ ____ ___ __**
2. **Please rate yourself in the following areas:**

| a. Please rate your **change in** **knowledge** in the following areas as a result of the workshop: | | | | |
| --- | --- | --- | --- | --- |
|  | **No change** | **A little better** | **Somewhat better** | **A lot more** |
| Choosing patient reported outcome measures (PROMS) |  |  |  |  |
| Developing a new PROM |  |  |  |  |
| Adapting PROMS in general |  |  |  |  |
| Cultural adaptation of PROMS |  |  |  |  |
| Testing for validity |  |  |  |  |
| Doing confirmatory or exploratory factor analysis |  |  |  |  |
| Writing a manuscript describing the process of PROM development or adaptation? |  |  |  |  |
|  |  |  |  |  |
| b. Please rate your **change in** **confidence** in the following areas as a result of the workshop: | | | | |
|  | **No change** | **A little better** | **Somewhat better** | **A lot more** |
| Choosing patient reported outcome measures (PROMS) |  |  |  |  |
| Developing a new PROM |  |  |  |  |
| Adapting PROMS in general |  |  |  |  |
| Cultural adaptation of PROMS |  |  |  |  |
| Testing for validity |  |  |  |  |
| Doing confirmatory or exploratory factor analysis |  |  |  |  |
| Writing a manuscript describing the process of PROM development or adaptation? |  |  |  |  |

For each question, choose the one correct answer.

**3. In a new setting, steps needed for using an existing PROM include:**

a) back and forward translations

b) assessing for semantic equivalence

c) cognitive interviewing

d) all of the above

e) none of the above

**4. In developing or adapting a measure, Confirmatory Factor Analysis (CFA) should be used:**

a) before PCA (principal components analysis) and EFA (exploratory factor analysis)
b) after PCA and before EFA

c) after PCA and EFA

d) the order does not matter

e) none of the above

**5. If two measures exhibit divergent validity, that means:**

a) the measures are highly correlated with each other

b) the measures are not very correlated with each other

c) one measure positively predicts the other measure at a future point in time

d) the measures represent all aspects of a given construct

e) none of the above

**6. In adapting a measure, cognitive interviewing is a:**

a) quantitative technique to assess reliability and validity of a measure

b) qualitative technique to assess expert opinions on semantic clarity of measure

c) quantitative technique to determine the underlying factor structure of a measure

d) qualitative technique to translate a measure from the source language to the target language

e) none of the above

**7. How successful was the workshop in meeting the goals of the workshop?**

- Not at all
- Somewhat
- A lot

**8. How successful was the workshop in meeting your goals of joining the workshop?**

- Not at all
- Somewhat
- A lot

1. **When did you feel most engaged in the workshop and why?**
2. **When did you feel the least engaged and why?**
3. **What 1-2 areas are of most interest for you for more learning in the future?**
4. **Are you thinking about incorporating any of the learning into possible /current research?**

- No
- Yes
- Not sure

**Please briefly explain your answer?**

1. If you are interested in other PCOR-related training and would like us to contact you, please include your email: ____________________
