# Supplementary material for: Building research capacity to adapt and develop Patient-Reported outcome measures in low- and middle-income countries: results from a psychometrics workshop in Tanzania
Source: BMC Health Serv Res. 2025 Jul 1;25:871. doi: 10.1186/s12913-025-13064-2 (PMC12219237; doi:10.1186/s12913-025-13064-2)
Supplement: Supplementary file 4 — Supplementary Material 4. [file 12913_2025_13064_MOESM4_ESM.docx]

**Supplementary Material 4**

**Table S1.**

*Comparison of pre- and post-workshop evaluations of reported knowledge and confidence*

|  | Knowledge | | | | |  | Confidence | | | | |
| --- | --- | --- | --- | --- | --- | --- | --- | --- | --- | --- | --- |
|  | M | SD | df | t | p |  | M | SD | df | t | p |
| PROMs Selection |  |  | 21 | -8.1 | <.001 |  | |  | 21 | -8.66 | <.001 |
| Pre-workshop | .82 | .91 |  |  |  |  | .91 | .97 |  |  |  |
| Post-workshop | 2.64 | .58 |  |  |  |  | 2.64 | .58 |  |  |  |
| PROMs Development |  |  | 20 | -11.61 | <.001 |  | |  | 20 | -13.71 | <.001 |
| Pre-workshop | .14 | .36 |  |  |  |  | .14 | .36 |  |  |  |
| Post-workshop | 2.43 | .68 |  |  |  |  | 2.24 | .63 |  |  |  |
| PROMs Adaptation |  |  | 20 | -10.18 | <.001 |  | |  | 21 | -10.65 | <.001 |
| Pre-workshop | .62 | .81 |  |  |  |  | .59 | .91 |  |  |  |
| Post-workshop | 2.71 | .46 |  |  |  |  | 2.73 | .55 |  |  |  |
| PROMs Cultural Adaptation |  |  | 21 | -10.68 | <.001 |  | |  | 21 | -12 | <.001 |
| Pre-workshop | .64 | .9 |  |  |  |  | .59 | .91 |  |  |  |
| Post-workshop | 2.82 | .4 |  |  |  |  | 2.77 | .43 |  |  |  |
| Testing for Validity |  |  | 20 | -9.64 | <.001 |  | |  | 21 | -10.65 | <.001 |
| Pre-workshop | .43 | .6 |  |  |  |  | .41 | .59 |  |  |  |
| Post-workshop | 2.48 | .6 |  |  |  |  | 2.5 | .6 |  |  |  |
| Testing for Factor Analysis |  |  | 20 | -13.39 | <.001 |  | |  | 20 | -13.36 | <.001 |
| Pre-workshop | .19 | .51 |  |  |  |  | .24 | .63 |  |  |  |
| Post-workshop | 2.38 | .59 |  |  |  |  | 2.52 | .51 |  |  |  |
| PROMs Manuscript Writing |  |  | 19 | -11.83 | <.001 |  | |  | 21 | -13.28 | <.001 |
| Pre-workshop | .25 | .64 |  |  |  |  | .41 | .67 |  |  |  |
| Post-workshop | 2.4 | .6 |  |  |  |  | 2.45 | .6 |  |  |  |
